# Anatomical variation in the form of inter- and intra-individual laterality of the calcaneofibular ligament

**DOI:** 10.1007/s12565-018-0440-3

**Published:** 2018-04-20

**Authors:** Hisayoshi Yoshizuka, Kentaro Shibata, Toyoko Asami, Akio Kuraoka

**Affiliations:** 1 Department of Anatomy and Physiology, Faculty of Medicine, Saga University, 5-1-1 Nabeshima, Saga, Saga 849-8501 Japan; 2 Department of Rehabilitation Medicine, Saga University Hospital, 5-1-1 Nabeshima, Saga, Saga 849-8501 Japan

**Keywords:** Anatomy, Ankle joint, Calcaneofibular ligament, Laterality, Variation

## Abstract

The lateral ligament complex of the ankle is involved in a large proportion of ankle sprains. The calcaneofibular ligament (CFL) is often involved in severe injuries. The purpose of this study was to evaluate the anatomical variation and laterality of the CFL to improve our understanding of the mechanisms of CFL-related injuries. This study utilized 110 paired ankles from 55 formalin-fixed Japanese cadavers (33 male and 22 female). The length and width of the CFL and the angle created by the CFL and long axis of the fibula (CF angle) were measured after exposing the CFL by careful dissection from the surrounding tissues. The results revealed that each parameter exhibited a wide range of values and showed unique patterns of frequency distribution, among which only the length was normally distributed. Among the parameters, only the CF angle showed no significant correlation with the other parameters. Analysis of laterality revealed that the mean left CF angle was significantly greater than the value on the opposite side (*p* < 0.05) and that the values of the bilateral CF angle showed no significant correlation at the individual level. The present results revealed not only detailed information regarding the CFL morphology, but also inter- and intra-individual laterality regarding the CFL traveling angle. It is likely that the differences in the quality and quantity of mechanical stress against each leg may have caused this morphologic laterality of the CFL.

## Introduction

**Fig. 1:**
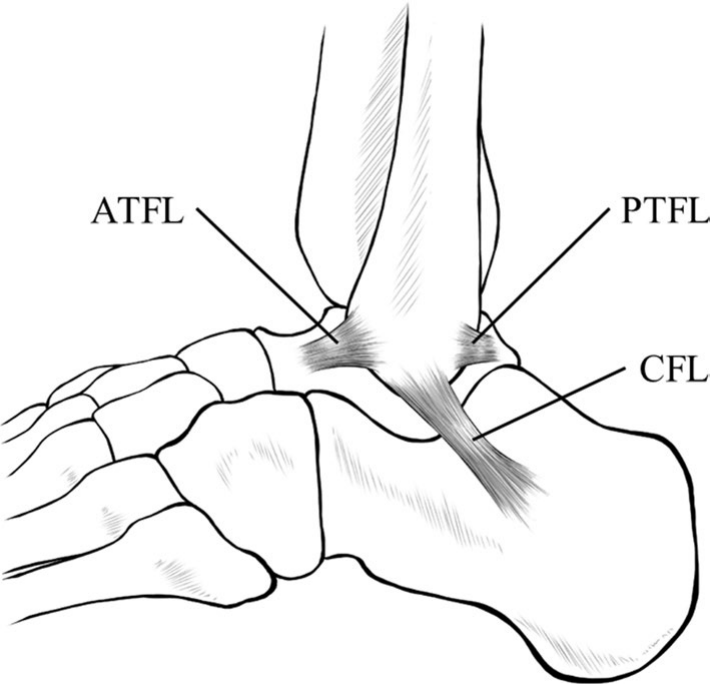
Schematic drawing of the lateral ligament complex of the ankle (left side). The lateral ligament complex of the ankle is composed of the anterior talofibular ligament (ATFL), posterior talofibular ligament (PTFL), and calcaneofibular ligament (CFL)

The lateral ligament complex of the ankle is composed of the anterior talofibular ligament (ATFL), posterior talofibular ligament (PTFL), and calcaneofibular ligament (CFL) (Fig. 1). Injuries to the complex, accounting for 85% of all ankle sprains (Chorley and Hergenroeder 1997), are the most common sports injury. Van Den Bekerom et al. (2008) noted that almost 80% of these injuries occur in the ATFL only, whereas the remainder are the result of combined damage of the ATFL and CFL. The authors also indicated that damage to the PTFL infrequently occurs only in cases of frank dislocation and that isolated injury of the CFL is rare. When the degrees of ligamentous damage and morbidity are taken into consideration, the injuries are classified as follows: grade 1 = partial rupture of the stretched ATFL, grade 2 = moderate injury including a complete tear of the ATFL and partial damage of the CFL, and grade 3 = complete disruption of both the ATFL and CFL (Ferran and Maffulli 2006). Thus, injuries involving the CFL tend to be severe, although the incidence of such injuries is relatively low. The presence of damage to the CFL affects not only the choice of operative or nonoperative treatment, but also prognosis. Seventy percent of patients with combined damage of the ATFL and CFL continue to complain of symptoms, such as pain and instability, after treatment (Samoto et al. 2007). Taken together, the CFL may be regarded as a clinically problematic ligament.

As reviewed by Matsui et al. (2017), several cadaveric studies have performed structural characterizations of the CFL using parameters such as the length, width, or size of attachment area. The results were variable even when comparable measuring methods were applied. For example, the length ranges from 19.5 mm (Milner and Soames 1998) to 31.94 mm (Taser et al. 2006). Another parameter, the traveling angle, which reflects the direction of the CFL, has been introduced in different definitions (Ruth 1961; Burks and Morgan 1994; Taser et al. 2006). The angle formed by the CFL and long axis of the fibula has been reported to range from 0 to 90° (Ruth 1961) and to show different distribution trends. Ruth (1961) reported that the rate of cases with this angle > 45° was 4.0%, whereas the same rate in another study was as high as 40.9% (Wiersma and Griffioen 1992). Thus, these indefinite trends in the CFL morphology suggest considerable variation in the characteristics of the ligament. However, strategies for assessing the ligament (e.g., the frequency distribution or normality test in larger sample sets) have not yet been developed.

Only Wiersma and Griffioen (1992) have referred to the laterality of the ATFL and CFL at the individual level. Briefly, using paired specimens, the scored findings of the ligaments were roughly compared between the right and left sides. Consequently, intra-individual variations of the ATFL and CFL were found in 33.3% (5 of 15 cases) and 66.6% (10 of 15 cases) of individuals, respectively. The authors mentioned that the results of the manual or roentgenographic stress examination of the ankle might be misunderstood because of intra-individual differences in the laterality of the ligaments. At present, however, the systemic or statistical analysis of laterality has not been performed with routinely employed parameters.

The purpose of this study was to evaluate variations of the CFL using an in-depth approach and in view of laterality to further the understanding of the mechanisms of CFL injury.

## Materials and methods

### Subjects

We assessed 110 paired ankles from 55 formalin-fixed Japanese cadavers (33 men and 22 women) used in a gross anatomy course conducted in 2014 and 2015 in the Faculty of Medicine, Saga University, Japan. In all cadavers, no traces of prior injury or surgical treatments were observed in the ankle region. The ages at the time of death ranged from 48 to 100 years [mean ± standard deviation (SD) 78 ± 12 years]. This study was approved by the local ethics committee of Saga University.

### Procedure of dissection

During the gross anatomy course, the bilateral lower legs of the cadavers were partly dissected. During this process, the skin was removed and the subcutaneous tissue, deep fascia, and muscles of the lower leg were exposed while the lateral ligaments of the ankle were left intact. First, we removed the peroneus longus and brevis tendons. Then, after observing the fiber directions, the CFL was dissected carefully from the surrounding tissues, including the peroneal tendon sheath, lateral talocalcaneal ligament (LTCL), and arch-forming fibers that unite the CFL and ATFL. The CFL was then assessed as follows.

### Measuring methods

**Fig. 2:**
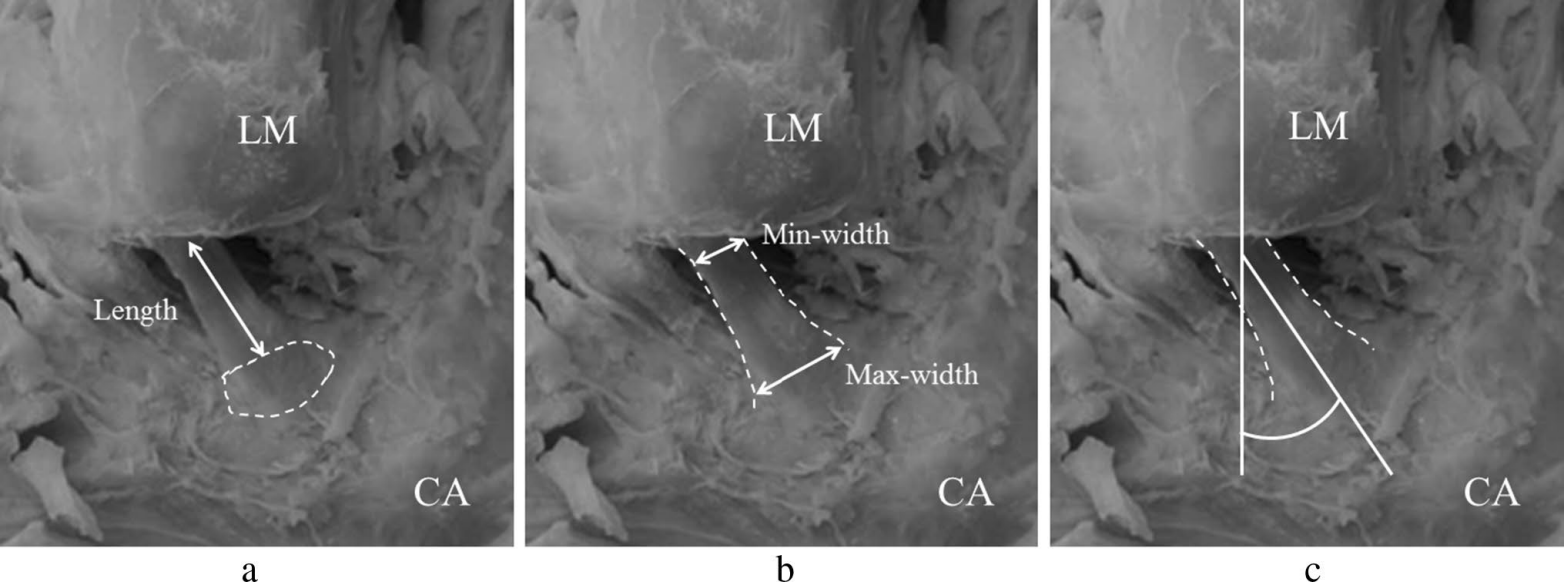
Measurement of the CFL (left side). The dashed line shows the margin of the attachment site (**a**) and lateral edge of the CFL (**b**, **c**). The perpendicular line indicates the long axis of the fibula (**c**). **a** The distance from the fibular origin to the most proximal insertion on the calcaneus (CA) defined the length. **b** Width was measured in two ways. **c** An angle was defined between the direction of the CFL and the long axis of the fibula and was designated as the CF angle. LM: lateral malleolus

The free length of the CFL was measured between the fibular origin and the most proximal insertion on the calcaneus (Milner and Soames 1998) (Fig. 2a). In this study, the maximum and minimum width (max and min width) was recorded because most CFLs were not constant in width (Fig. 2b). As for angular parameters, the angle created by the CFL and long axis of the fibula (CF angle) was measured according to the method of Ruth (1961) (Fig. 2c). The fibular long axis was defined by connecting two midpoints set by measuring the width of any region at the diaphysis.

All measurements were performed by a single researcher (H.Y.) by setting both the talocrural and subtalar joints in the 0° position and using a caliper (Fujiwara Sangyo, Hyogo, Japan) or goniometer (OG Wellness Technologies, Okayama, Japan) on each CFL. A minimum unit of records was defined as 0.5 mm for distance or 5° for angle. The accuracy of the measurement technique was determined by repeating all measurements in technical triplicates. The findings in all specimens were photographed using a NEX-5R digital camera (Sony, Tokyo, Japan) for future reference.

### Statistical analysis

All statistical analyses were performed using IBM SPSS Statistics version 22, and the level of statistical significance was set at a *p* value of 0.05. The Shapiro-Wilk test was used to evaluate normality. Laterality between grouped data from each side (inter-individual laterality) was examined by a paired *t* test or Wilcoxon signed-rank test. Pearson’s correlation coefficient or Spearman’s rank correlation coefficient was calculated to investigate laterality at the individual levels (intra-individual laterality) and the relationships among parameters.

## Results

During sample preparation, we noticed a cadaver (64 years of age, male) in which the left CFL was deficient and excluded it from the study. Therefore, data from 108 paired ankles from 54 cadavers (32 male and 22 female; mean ± SD: 78 ± 12 years of age) were included in the subsequent analysis.

### Anatomical variation of the CFL

Table 1 shows the mean values and range of measured CFL dimensions. All parameters exhibited a wide range, and the ratio of the maximum to minimum value was 2.7, 4.0, and 3.4 for the length, max width, and min width, respectively. In most CFLs, the max and min widths were located near the fibular origin and the calcaneal insertion, respectively (Fig. 2).

**Table 1:** Dimensions of the calcaneofibular ligament (*n* = 108)

	Mean ± SD	Range
Length (mm)	17.7 ± 3.5	11.0–30.0
Max width (mm)	8.9 ± 2.1	3.5–14.0
Min width (mm)	4.9 ± 1.0	2.5–8.5
CF angle (°)^a^	24.3 ± 15.1	0–70

^a^CF angle = angle created by the calcaneofibular ligament and long axis of the fibula

**Fig. 3:**
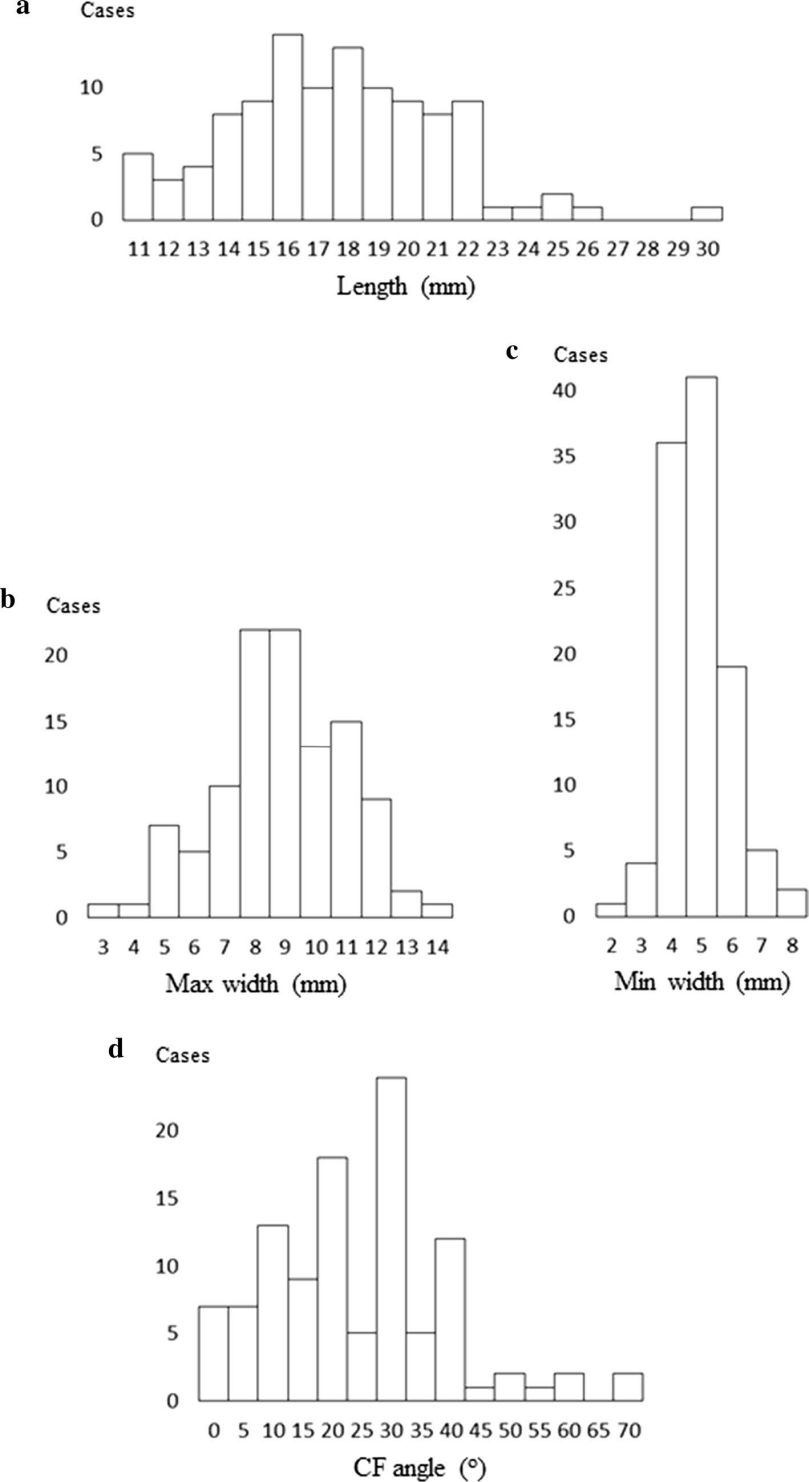
Histogram showing CFL dimensions (*n* = 108). For convenience, two successive minimum units were combined for length and width and expressed in one bar. For example, a bar showed 11 mm for length, including values of 11.0 and 11.5 mm

The frequency distributions of the CFL dimensions are shown in Fig. 3. The length values seemed to be distributed with the characteristics of a relatively low peak, gentle curvature, and a tendency toward lacking values > 23 mm (Fig. 3a). In contrast, the peak frequency for each width was apparent (Fig. 3b, c). Most samples were distributed within a narrow range (i.e., 8.0–11.5 mm for max width and 4.0–6.5 mm for min width). The frequency distribution of the CF angle appeared to be biased (Fig. 3d). The total number of cases with an angle < 40° accounted for as much as 92.6%, whereas the remainder had angles evenly scattered within a range of 45–70°. Examinations for normality revealed that only length was normally distributed (*p* = 0.08).

To examine the relationships among parameters, correlation coefficients were calculated (Table 2). Among parameters indicating distance, moderate or weak positive correlations were calculated except for the combination of length and min width. The CF angle was not significantly correlated with other parameters.

**Table 2:** Correlation coefficients of calcaneofibular ligament (CFL) morphology

	Length	Max width	Min width	CF angle^a^
Length		0.23*	n.s.	n.s.
Max width			0.51***	n.s.
Min width				n.s.
CF angle				

*n.s.* not significant

*p < 0.05, ***p < 0.001

^a^CF angle = angle created by the CFL and long axis of the fibula

### Analysis of laterality

The collected data from each side were compared to examine inter-individual laterality (Table 3). Among the parameters, only the left CF angle was significantly greater than that on the right side (*p* < 0.05).

**Table 3:** Statistical analysis of inter-individual laterality

	Right (*n* = 54) Mean ± SD	Left (*n* = 54) Mean ± SD	*p* value
Length (mm)	17.5 ± 3.5	17.9 ± 3.6	0.34
Max width (mm)	8.6 ± 2.1	9.2 ± 2.1	0.06
Min width (mm)	4.9 ± 1.2	5.0 ± 0.9	0.36
CF angle (°)^a^	20.9 ± 13.8	27.6 ± 15.7	< 0.05

^a^CF angle = angle created by the calcaneofibular ligament and long axis of the fibula

**Fig. 4:**
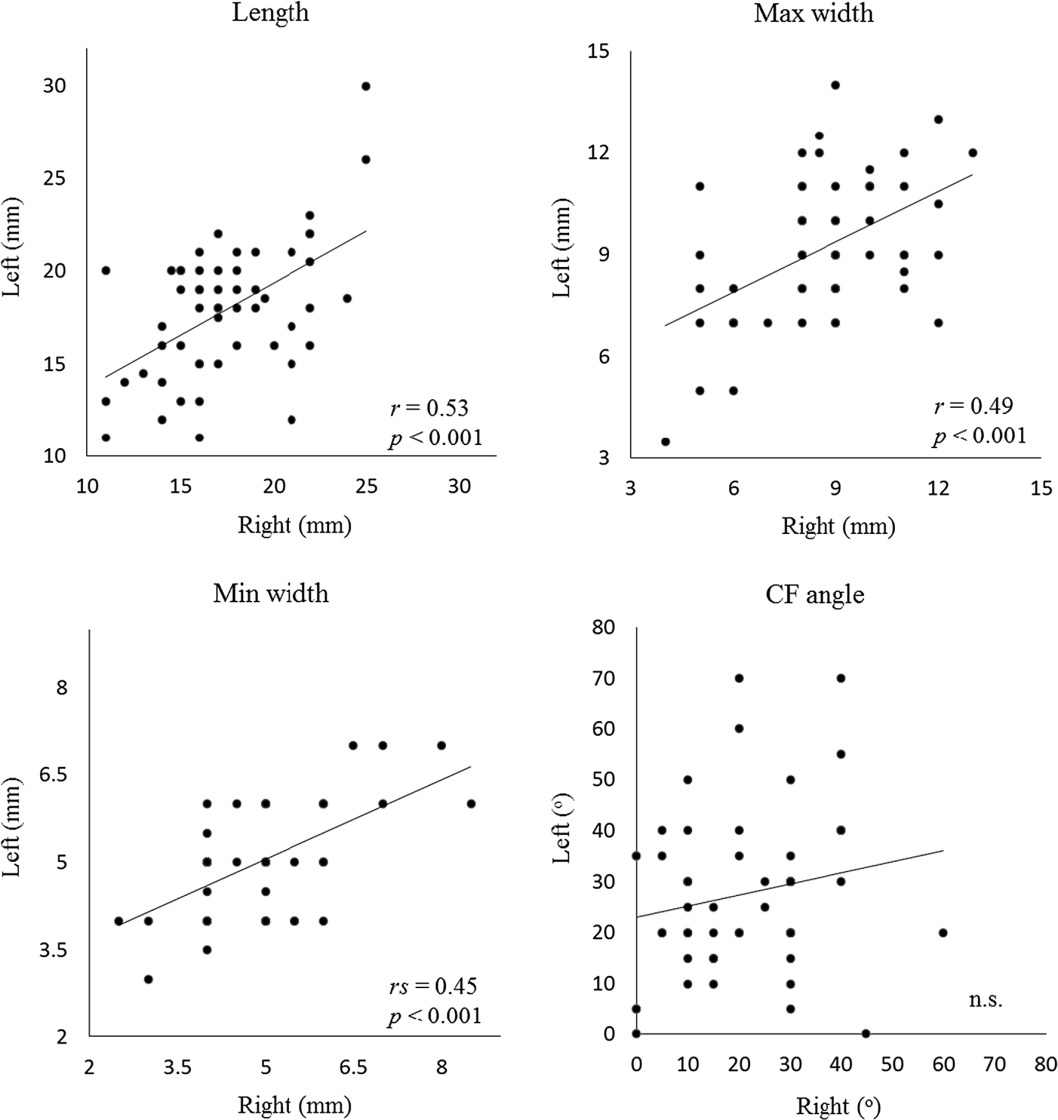
Scatter plot of morphologic parameters within individuals (*n* = 108). All approximately straight lines are shown as the eye guide. Only the CF angle exhibited no significant correlation, whereas the rest had moderate positive correlation

To visualize and examine intra-individual laterality for each parameter, we prepared scatter plot diagrams and calculated correlation coefficients between the two sides (Fig. 4). There was a moderate positive correlation for length, max width, and min width. In contrast, only the CF angle showed no significant correlation.

## Discussion

The mean CFL length obtained in this study was similar to the value reported by Milner and Soames (1998) (17.7 ± 3.5 vs. 19.5 ± 3.9 mm). The mean maximum width (8.9 ± 2.1 mm) and minimum width (4.9 ± 1.0 mm) were also close to values corresponding to the distal end of the calcaneal insertion (9.68 ± 1.73 mm) (Taser et al. 2006) and midpoint (4.68–6.7 mm) (Ruth 1961; Buzzi et al. 1993; Taser et al. 2006; Yildiz and Yalcin 2013), respectively. However, the CF angle showed not only a somewhat limited range (0°–70° vs. 0–90°) (Ruth 1961), but also a smaller mean value (24.3° ± 15.1° vs. 43°–47°) (Burks and Morgan 1994; Yildiz and Yalcin 2013). Thus, for the CF angle only, differences were noted when comparing the value with the results of previous studies. Furthermore, the mean left CF angle was significantly greater than the angle on the opposite side (*p* < 0.05) (Table 3), and the scatter plot diagram demonstrated that the values of the bilateral CF angle showed no significant correlation at the individual level (Fig. 4). These results suggest the presence of inter- and intra-individual laterality of the CFL traveling angle. The characteristics of the angle may be caused by ethnic differences. However, laterality may be related to handedness and footedness, which are not restricted to Japanese people. Reportedly, right-handers clearly show a right-foot bias for activities requiring fine manipulation and focused attention (Peters 1988). In addition, from a developmental perspective, only 5% of juveniles indicate a left-foot preference (Gentry and Gabbard 1995). Thus, it is reasonable to suggest that the left leg exerts a supportive role in many people. A tendency to have a longer and heavier left leg in adult right-handers has also been demonstrated (Peters 1988). Considering normal activities of daily life and sports activities, the CFL of the supporting leg should have more opportunities to brake the motion of the lower leg when the foot is under load. Furthermore, the mean left maximum width was also greater than that on the right side (9.2 ± 2.1 vs. 8.6 ± 2.1 mm), and this difference approached significance in a paired *t* test (*p* = 0.06) (Table 3). As the maximum width reflects the size of the calcaneal insertion, it is logical to suppose that the quality and quantity of mechanical stress differ between the two sides. Such differences may have affected the onset of morphologic laterality of the CFL observed in this study.

The present results revealed a wide range and unique pattern of frequency distribution for each parameter (Fig. 3), among which only length showed a normal distribution. In particular, the CF angle showed no significant correlation with other parameters (Table 2). Sarrafian and Kelikian (2011) mentioned that the position in which the CFL is tight differs among individuals in cadaveric studies. These results indicate that not only morphologic variation of the CFL, but also the possibility that the variation may influence susceptibility to or severity of injury and represent one cause of an isolated injury of the CFL. In addition, the CFL and ATFL are often united by arch-forming fibers and work as a functional unit to stabilize the ankle (Yildiz and Yalcin 2013). In some cases, even deficits of the ATFL have been noted (Kitsoulis et al. 2011; Raheem and O’Brien 2011). These factors further complicate the situation. However, as the ATFL, PTFL, and CFL function synergistically to stabilize the foot, variation in any ligament impacts the other ligaments. Thus, we hypothesized that such variations may represent the complementary function of the ligaments. Although no research has examined the components of the lateral ligament complex simultaneously in each ankle, this approach might be useful for identifying the significance of CF angular variation.

It has been previously documented that the CFL crosses over the deep side of the peroneus longus and brevis tendons. In this study, we confirmed this structural relationship in all 108 samples. Considering this positional proximity, Shinohara et al. (2007) speculated that both tendons were lifted by the tensed CFL during ankle inversion. Although this phenomenon has not been demonstrated experimentally in living bodies or cadavers, it suggests that the CFL works as a tensioner of the tendons to control ankle joint stability by enhancing muscle contraction. In general, braking lower leg movement by eccentric or isometric contractions of the peroneus longus and brevis muscles is indispensable in the weight-bearing position. According to this hypothesis, the tensed CFL will maximize the braking effect in response to occasional weight shifting, which forces the foot from the neutral to inverted position. Furthermore, variation of the CFL may cause differences in the efficiency of the effect at the individual level and excessive lift-up may cause peroneal tendon dislocation. Thus, this hypothesis is quite attractive from both the functional and clinical perspectives. Further biomechanical studies are required to test this hypothesis.

Burks and Morgan (1994) described a type of LTCL that is barely distinguishable from the CFL, although the incidence of this entity has not been confirmed. We encountered several cases in which the LTCL ran parallel to and was fused with the CFL while dissecting our 108 specimens, which rendered such dissections quite challenging. Thus, considerable attention will be required during the morphometric evaluation of the CFL using magnetic resonance imaging or other diagnostic imaging modalities.

In the present study, as information on handedness of the cadavers was unavailable, we could not analyze the laterality of the CFL by separating them into right- and left-handed individuals. This is a limitation of this study.

In conclusion, the present results revealed not only detailed information regarding CFL morphology, but also inter- and intra-individual laterality regarding the CFL traveling angle. It is likely that the differences in the quality and quantity of mechanical stress against each leg may have caused this morphologic laterality of the CFL.
