# Correction to: Anatomical variation in the form of inter‑ and intra‑individual laterality of the calcaneofibular ligament

**DOI:** 10.1007/s12565-021-00617-8

**Published:** 2021-05-27

**Authors:** Hisayoshi Yoshizuka, Kentaro Shibata, Toyoko Asami, Akio Kuraoka

**Affiliations:** 1 Department of Anatomy and Physiology, Faculty of Medicine, Saga University, 5‑1‑1 Nabeshima, Saga, Saga 849‑8501 Japan; 2 Department of Rehabilitation Medicine, Saga University Hospital, 5‑1‑1 Nabeshima, Saga, Saga 849‑8501 Japan

## Correction to: Anatomical Science International (2018) 93:495–501 10.1007/s12565-018-0440-3

The article “Anatomical variation in the form of inter‑ and intra‑individual laterality of the calcaneofibular ligament”, written by Hisayoshi Yoshizuka, Kentaro Shibata, Toyoko Asami, Akio Kuraoka, was originally published Online First without Open Access. After publication in volume 93, issue 4, page 495–501 the author decided to opt for Open Choice and to make the article an Open Access publication. Therefore, the copyright of the article has been changed to © The Author(s) and the article is forthwith distributed under the terms of the Creative Commons Attribution 4.0 International License, which permits use, sharing, adaptation, distribution and reproduction in any medium or format, as long as you give appropriate credit to the original author(s) and the source, provide a link to the Creative Commons licence, and indicate if changes were made.

The images or other third party material in this article are included in the article’s Creative Commons licence, unless indicated otherwise in a credit line to the material. If material is not included in the article’s Creative Commons licence and your intended use is not permitted by statutory regulation or exceeds the permitted use, you will need to obtain permission directly from the copyright holder.

To view a copy of this licence, visit http://creativecommons.org/licenses/by/4.0/

The original article has been updated.

